# Local Environmental Grassroots Activism: Contributions from Environmental Psychology, Sociology and Politics

**DOI:** 10.3390/bs5010121

**Published:** 2015-03-23

**Authors:** Nikolay L. Mihaylov, Douglas D. Perkins

**Affiliations:** Department of Human and Organizational Development, Peabody College, Vanderbilt University, Box 90, 230 Appleton Place, Nashville, TN 37203, USA; E-Mail: d.perkins@vanderbilt.edu

**Keywords:** local activism, grassroots activism, environmental activism, social movements, environmental psychology, place attachment, social representations

## Abstract

Local environmental grassroots activism is robust and globally ubiquitous despite the ebbs and flows of the general environmental movement. In this review we synthesize social movement, environmental politics, and environmental psychology literatures to answer the following questions: How does the environment emerge as a topic for community action and how a particular environmental discourse (preservation, conservation, public health, Deep Ecology, justice, localism and other responses to modernization and development) becomes dominant? How does a community coalesce around the environmental issue and its particular framing? What is the relationship between local and supralocal (regional, national, global) activism? We contrast “Not in My Back Yard” (NIMBY) activism and environmental liberation and discuss the significance of local knowledge and scale, nature as an issue for activism, place attachment and its disruption, and place-based power inequalities. Environmental psychology contributions to established scholarship on environmental activism are proposed: the components of place attachment are conceptualized in novel ways and a continuous dweller and activist place attachment is elaborated.

## 1. Introduction

Public concern over toxic contamination of our air, water, land and food and the startling loss of natural beauty and resources to development rose sharply in the 1960s and 1970s. Despite subsequent government and corporate responses, continued concerns over various environmental threats have led to a vast and growing environmental movement of activists, supporters, organizations and members. By 2000, there were well over 6000 national and regional environmental movement organizations in the United States alone, and more than 20,000 local organizations [1] and with increased concern over global climate change, the number of grassroots and professionally-staffed environmental advocacy organizations worldwide has risen sharply. Entire, environmentally-focused branches of sociology and political science have also grown in rough proportion to those public concerns and organized responses. In contrast, until recently psychologists have devoted relatively limited attention to perceptions, cognitions, attitudes, emotions, motivations and behaviors in response to environmental concerns, and what they have studied is mainly at the individual level, largely ignoring the critical ecological context and collective psychology and behavior of environmental activism.

Our goal is to place the psychology of environmental activism in the broader context of the sociological, political and environmental studies literatures on environmental social movements. We want environmental psychology to realize its vital relevance and importance on this issue and fulfill its potential in helping us understand both individuals’ *and* communities’ complex responses to collective environmental threats. To this end, we review sociology and environmental politics research on local environmental activism, point out blind spots or directions for expansion, demonstrate the usefulness of place-based concepts, relate place attachment research to studies of activism, and propose theoretical syntheses and developments that can serve future studies. We begin by reviewing the history of, and prior research on, grassroots environmental activism, including the origins of environmentalism, New Environmentalism, and the environmental justice movement. Our attention then turns to the increasing significance of the local scale in contentious politics and pose our three guiding questions for the review and theory development of local environmental activism: (1) How does the environment emerge as a topic for community activism and which environmental discourse (e.g., preservation, conservation, justice, health, *etc*.) emerges as dominant? (2) How does a community become consolidated around the environmental issue and its particular framing? (3) What is the relationship between local and supralocal (regional, national, global) activism? To address these questions, we first turn to prior research, then describe the key characteristics of the local, the environment, and their interaction in place and local knowledge, place-based power inequalities and vulnerability. The latter half of the article addresses the three central questions in some depth, synthesizing our three main scholarship sources and proposing new directions for research. The last section contrasts NIMBY and more globally focused and liberatory forms of environmental activism. We conclude with a brief reflection on place identity and inequality, human agency and community empowerment.

## 2. History of Grassroots Environmental Activism

### 2.1. Traditional Environmentalism: Preservation and Conservation

The environmental movement emerged in the second half of the 19th century. From its very start two environmental perspectives, embodied in the debates between John Muir and Gifford Pinchot, competed and complemented each other: preservation and conservation [2]. Preservation efforts were directed toward keeping parts of the natural world, seen apart from human society and with an intrinsic value, undisturbed by industry, often for human purposes like recreation. Conservation had a clear utilitarian concern: its purpose was to ensure the sustainable use of a natural resource for generations to come. Both of these environmental *discourses* [2] were reactions to the effects of early capitalism; they were, however, centered around similar assumptions of modernity and utilitarianism that brought about the industrial revolution and its environmental consequences in the first place. These assumptions were the domination of nature by man and an anthropocentric view of life where nature was an object of human needs for survival or thriving [3]. This period of “Romantic Environmentalism” [4] lasted well into the 1950s, but preservation and conservation are still among the most legitimate and widely-used discourses and strategies for addressing environmental issues.

### 2.2. New Environmentalism

The 1960s brought an “environmental revolution” [5] in Western societies. Environmental issues came to the front of public attention with some well-publicized ecological disasters and popular books like Rachel Carson’s *Silent Spring* [6]. With the post-WWII economic growth, industrialization, and urbanization came quantitative and qualitative increases in environmental disruption and risk, and hence the reckoning that environmental issues were human health issues, and that humans are not apart from nature; a response to ecological threats to human health was necessary [2]. The new health concerns were perceived by a new audience of a well-off middle class with increasingly post-materialist values [7], in an atmosphere of general support for social change and social movements [8]. Conservation and preservation were seen by many as no longer adequate to address the inseparable relationship between human life and nature. Thus a mass movement of “new” or “reform” environmentalism [2] emerged. This environmental movement shares important themes with new social movements (NSMs) [7], namely post-materialist values (with questioning of economic growth), and the promotion of autonomy, self-determination and oppositional collective identity (with the call for a green lifestyle and independence from governmental or corporate control). The movement produced “green politics”, especially in Europe, where Green parties came to represent environmental concerns in parliaments. Environmental protection was also institutionalized in the United States, in direct response to severe water and air pollution, through bodies like the Environmental Protection Agency.

A more radical shift in thinking about the relationship between humans and nature emerged in this period: the Deep Ecology perspective [9]. Deep Ecology stood for equality for all forms of life, thus stepping away from anthropocentrism. Every human generation should pass the world on to the next in the same condition they received it. This idea was directed more to the everyday thinking and way of life of humans than to structural forces for environmental issues. Thus, one of the main criticisms toward Deep Ecology was that some of its proponents put the blame on human nature, in an individualistic framework of explanation.

### 2.3. Environmental Justice Movement

New Environmentalism was critiqued from the political right and left. It was also curbed institutionally during the 1980s when neoliberal administrations started to ascend to power in the West. On the one hand, the economic hardships of the 1970s brought back growth and capital accumulation as highest state priority [10]. This comeback made national-level policy reforms aimed at restrictions on corporate pollution harder to promote. On the other hand, the expansion of environmental policy made apparent the differential treatment of certain groups and communities when protection of nature was enforced in practice by institutions. New Environmentalism reflected the transformation of the environment into a mainstream issue. The Environmental Justice Movement (EJM), the next wave of environmental activism [4] reflected the realization that humans are indeed part of nature, but there are social differences among them modifying that relationship. Progressive environmental reform made big strides to protecting human health and nature; but social inequalities meant that White middle-class citizens were the main group that enjoyed these new environmental rights. One of the goals of EJM was to expand these rights to all groups in society, including those with less power and a history of discrimination.

EJM emerged from the experiences of newcomers to the environmental movement: marginalized social groups living in polluted communities sacrificed for economic growth. Central in these groups’ experiences were themes of injustice, deprivation and discrimination, and struggles for self-determination and land sovereignty [4]. To define environmental justice from a community perspective, Schlosberg [11] proposes three equally important components: equity in the distribution of environmental risk, recognition of the diversity of the participants and experiences in affected communities, and participation in the political processes which create and manage environmental policy.

While the policy-level expansion of environmental protection was less likely in the new pro-growth climate, the horizontal expansion of environmental rights to local communities proved a more viable path for environmentalism. One other key factor for shaping EJM should be noted: the legacy of the civil rights movement provided experience in activism and, perhaps even more importantly, a rights master frame [4,12]. Community struggles for healthy environment were justified as a seamless extension of the struggle for rights—this time rights to clean water, air, food. To summarize, the most significant feature and contribution of EJM is the assertion of a strong link between social justice and nature. It is a two-way relationship: protecting the environment is a social justice activity because marginalized communities are hit hardest by pollution; promoting social justice helps the environment because it is the social, economic and power marginalization of communities that opens weak spots in the enforcement of environmental protection.

The EJM is much more “local” than policy-level challenges and this localism begets some new and different characteristics of activism. First, because a marginalized community faces multiple issues of discrimination—social, employment, investment, housing, education, *etc*., environmental justice is one topic in the agenda of multi-issue organizing [13]. Indeed, in the real-life experiences of marginalized communities, environmental injustices are not abstractly separated from other domains of discrimination. Most often people in poor communities reside, work, learn, and take leisure within the spatial boundaries of their community [4]. This holistic experience and multi-issue activism makes the study of environmental efforts harder, because they are intertwined with other goals and actions. Second, while there are multiple possible bases of power inequality, race, class or ethnicity are the dimensions that are most likely to be quite homogeneous at the level of the community, where environmental threat or resistance happens. Third, there are complex relationships between local and national or policy-level environmental activism. Some scholars and activists call EJM a grassroots movement [14,15], or “the new grassroots” [16], more radical in its empowerment of the victims of economic and power domination. Localism can also be seen as limiting, because local wins do not change environmental policy; local groups fight for enforcement, not reform [14]. This focus can be interpreted as parochial, or Not-In-My-Backyard (NIMBY) activism. Of course, local grassroots environmental groups should not be subsumed under one type. Solidarity with other groups pays off as cooperation increases the political leverage of protest activities [12]. There is also a complex two-way relationship between local and national levels of environmental activism: local groups discover patterns to their grievances related to discrimination, while national groups educate local groups in the environmental justice framing [12]. Some division of labor is also evident, for example direct action on local level and litigation on national level of organizations [4].

The main battlefield of EJM might be local communities, but the power inequalities and their social bases revealed by this struggle have helped the advancement of environmental thought as well. Once highlighted, the link between nature and power has been examined from multiple viewpoints. Instead of assuming a polar relationship between human society and nature, “political ecology” [17] examines a dynamic relationship between them structured around different power axes. Some of these axes are modernity, class, and race.

One important source of power inequality stems from modernization. The project of modernity is about making the world legible and manageable [18]. It entails measurement, standardization, and administrative ordering of nature and society, based on rationalization, bureaucratization, and the application of science and technology. This vision is promoted by an ideology of progress, where some worlds (social organizations, economic forms, styles of administration, and ways of life) are more effective and civil than others and must replace them [19]. Modernization, the process of replacing the traditional worlds with the modern, is driven by the state and the business organization. Outcomes of modernization are industrialization, urbanization, a consolidated state, a capital-accumulation economy, and more recently, globalization and surveillance. Modernization can be served by overt military force, as in colonial exploitation, or a cultural myth of progress, as in peripheral regions of the West itself [19]. The discourse of “development” is seen by many as another chapter in the expansion of Western reason [17].

Modernization creates a conflict between system and lifeworld [20]. The power inequality stems from the coercive capacities of the state and organized capital, and the cultural appeal of the myth of progress. Resistance to modernization can be a throwback to pre-modern life, or a post-modern libertarian alternative. One important field of struggle is between colonial exploitation and indigenous people, the movement for indigenous rights. A romanticized, early framing of indigenous rights affirmed local knowledge of (harmonious) natural resources use and local ways of life as an alternative to (industrial) development. A more realistic picture is for “an indigenous, grassroots-controlled modernization” [21], *i.e.*, for locally-controlled development. Within Western societies, contentious rural politics exemplifies the conflict along this axis [22]. As rural communities reject the role of resource extraction periphery in a rationally-ordered economy, they engage in both identity-based challenge to the cultural myth of modernization and a political struggle with state power for local control [22]. A reactive, pre-modern response claims the preservation of (or return to) a pre-industrial, pastoral communal life. A more pragmatic, conservation approach is observed when communities defend investments in commodified ruralism (e.g., tourism) or lifestyle (as in wildlife management by hunters). Finally, a post-modern alternative stems from contested rural identity as a basis for a new social movement. This response emphasizes self-sufficiency and autonomy, and a simple, close-to-nature, interpersonal-solidarity rural society. All of these responses share a mix of pre-modern and post-modern rejection of scientific expertise and the interfering state (in a characteristic NSM negative framing of demands [23]) coupled with an affirmation of local knowledge, self-determination, and more democratic, local control. This discourse can happen within an environmental rights framing, as with the demands for local empowerment and determination [24], but also within conservation, preservation, health or even deep ecology framing. 

Ecological Marxism [25] links class and political economy with ecology. In this development of Marxist thought the exploitation of nature and labor are closely related as they are production conditions which capital cannot produce itself as commodities for further production. This pressure on natural resources from capital accumulation is augmented by the exploitation of the labor of poor people around the world. And poverty has been established as a major determinant of ecological degradation [17], as the discussion below on vulnerability shows.

Finally, race has been studied as the most significant dimension in the social production of environmental inequalities [26]. Environmental racism is the racial discrimination in environmental policy-making, policy implementation, and decisions with regard to the siting of risky or controversial facilities [26]. The EJM is often described as the people of color environmentalism [4], as it emerged in urban communities of color where power inequities and marginalization were facilitative of projects like landfills and pollution industries. 

To summarize, the critical awareness of power and power inequities around environmental issues is what makes EJM different from previous waves in environmentalism. Since the 1990s environmental efforts have been directed along the discourses that emerged from the three waves of environmentalism. First, the established institutional approaches of conservation and preservation, exemplified by organizations such as Sierra Club and the Nature Conservancy. Second, New/Reform Environmentalism with its focus on human health and ecological threat, represented by organizations such as Greenpeace and Friends of the Earth [27]. And third, the Environmental Justice Movement with its critical awareness of power and a strong grassroots base [28].

## 3. The Increasing Significance of the Local Scale

Does the arc of history of environmentalism bend to the local? The EJM narrative is clearly about bottom-up activism, the “new grassroots” [15,16], trying to remedy the shortcomings of previous waves of environmentalism [26]. Evaluations of the EJM see its clearest victories at the local level [26]. More importantly, local environmental activism is “ubiquitous and recurrent, even in times when environmental issues are not salient on [the] national agenda” [29] (p. 722). Some studies suggest a dramatic increase in the number of local environmental groups in the 1990s and 2000s [30]. What contributes to the localization of environmental activism?

Firstly, the logic of environmental politics in the last decades has been about localizing. There are two main reasons for this. On the one hand, after the significant policy wins of New/Reform Environmentalism, implementation became the contested stage of the policy-making cycle. And, as Rootes [29] points out, “implementation of environmental policy is necessarily local; the local is where the rubber of policy meets the road of obdurate local circumstances” [29] (p. 733). On the other hand, there is a consistent trend in environmental politics and activism to recognize and involve new groups and communities. If early environmentalism was a cause of a small elite (Muir and Pinchot), new environmentalism became a mass movement of the White middle class, and the EJM involved hitherto marginalized communities [31]. This expansion was coupled with an increasingly differentiating view of humans and nature: from humans apart from nature to humans within the environment, and finally, to the realization that humans are not equal with regard to their environmental conditions and outcomes. Local communities therefore are important agents and contexts of environmental politics.

Secondly, the political and economic logic of neoliberal capitalism increases the importance of the local scale of development. Capitalism produces uneven geographical development in all of its phases, as some places are “systematically privileged over and against others as sites for capital accumulation” [32] (p. 355). With the advent of neoliberalism the polarizations between localities became especially strong, because the stabilizing role of the Fordist-Keynesian state was diminished, along with the import of its national scale of regulatory planning, decision-making and policy implementation [32,33]. In neoliberal governance local communities are increasingly responsible for local development and services (“governing through communities”, [22])—both with regard to decision-making and resource provision [22,34,35]; they become the basic economic unit where political struggles and socioeconomic production take place [32]. The result is commodification of the local [22] and competition between localities in a constant struggle for being core and not periphery, and for the very definition of these positions. The uneven distribution of gains and risks [26] among localities is directly related to the environment: some localities become collectors for others’ refuses from development; some define, hoard or exploit valuable resources; yet others turn the environment into a quality-of-life selling point for desired dwellers.

Thirdly, from a sociological perspective, there is the trend of shifting politics to more particularistic contexts, from national to community and individual scales. New social movements theory describes the decentralized, expressive and identity-based protest in post-modern society [7]. Local environmental grassroots activism can be explored through NSM lenses because contentious politics of a locality often possesses some or all of these characteristics: it is based on or in defense of a shared place identity; it is submerged in residents’ everyday lives and interpersonal networks within a place; it is reactive to the intrusion of outside state or corporate forces; and it is particularistic, often turned inward to a self-defined way of life.

The focus on the local scale of environmentalism should be accompanied with a critical examination of environmentalism’s history narrative. Brulle and Pellow [26] observe that the literature is uncritical and quite celebratory of the EJM. A frequent practice in the study of EJM is to assume that the community is a single agent, a unitary unit; within-community differences and conflicts over environmental issues are neglected (e.g., [11,13]). This blind spot is exemplified well in Schlosberg’s [11] expansion of the environmental justice concept to include recognition and respect of the diversity of experiences in affected communities, where he means *of* affected communities, not *within* them. Hence often the result is what Saitta [19] criticizes as research of movements in areas of risk focused on local elites—“active, informed, and reflective citizens (environmental movements, boards of citizens, and the like)” rather than on “ordinary people” and the general ambivalence of the inhabitants. Thus, the focus on the local should serve both an intellectual and an ethical purpose—to advance the logic of environmentalism’s recognition and inclusion of groups—now within communities (and especially the non-elites), and to uphold the principle of justice by respecting diversity within communities. The question of how the environment emerges as a (common) community issue is worth exploring in itself.

Despite the presentist bias of progress in the history of the environmental movement, in actuality most environmental discourses coexist today [2], including within the missions of leading environmental organizations: preservation, conservation, health and risks, deep ecology, environmental rights and justice, local control and indigenous rights, local *vs*. expert knowledge, and the NSM “leave us alone” motto. These discourses are in complex relationship, competing with and facilitating each other [1]. Even at the local level, environmental justice is not necessarily the dominant discourse, as Andrews and his colleagues show in their studies [36,37] of North Carolina’s local environmental groups [38]. How a particular environmental discourse becomes dominant in a local grassroots mobilization is another question to study as we focus on the local.

Based on this review and analysis of environmentalism’s history, we will structure the rest of our review around three groups of questions that we think capture the emergence and development of local environmental activism:

1. How does the environment emerge as a topic for community activism? What environmental discourse emerges as dominant in a community and how?
2. How does a community become consolidated around the environmental issue and its particular framing?
3. What is the relationship between local and supralocal activism (regional, national, global)?

## 4. Prior Research on Local Environmental Grassroots Activism

We can draw on several research disciplines and bodies of literature to address these questions. A major source is social movement or, more generally, contentious politics [39] studies. Local protest organizations are understudied in the social movements field [37], and even less is grassroots environmental activism [29]. In their review of studies of threat as an impetus for mobilization, Johnson and Frickel [40] do not cite a single study of environmental threat; this is surprising given the fact that environmental activism, and especially its local forms, is mostly a reactive movement [40,41].

Research from a SM perspective is focused on established topics such as participation in local environmental organizations [42], organizational characteristics [37], selection of tactics [43], environmental coalitions [44], political opportunities and outcomes [10], the media [36], and SM sector determinants of the founding of environmental organizations [1,45]. This body of research, while illuminative, is mostly focused on the organizational and supra-organizational level of activism, which leaves untouched important aspects of local grassroots activism. A branch of contentious politics studies that addresses more squarely the local level is environmental politics. An issue of the eponymous journal showcased studies of the organizational links of local to national activism, links between place and contention, and framing of local environmental protests [29].

The first two questions we posed for examination above reflect the need we see to focus on not just predictor variables, but also on the social and cognitive processes of local environmental activism. Somewhat unexpectedly, a handful of rural studies [34,35] examine the links between the construction of place meaning, local activist identity, and mobilization, by developing the concept of “place-framing” (after “framing processes” in SM studies [46]). We welcome this cross-pollination between SM and geographic studies and will propose suggestions for advancing this approach through the use of environmental psychology research. Place attachment theory [47] applies concepts such as sense of place and place identity to examine links between individuals, communities, and their physical environment. One of the major topics in this body of research is the links between place attachment and place symbolic meanings, and inhabitants’ reactions to disruptions and changes in the place (see [48] for a review). This focus makes place attachment theory especially relevant to the study local environmental activism, and its hereby proposed collaboration with contentious politics research—promisingly fruitful. Recently, environmental psychology has adopted a social representations approach [48,49] that has similarities to the framing processes concept in SM research; thus both perspectives (hopefully knowing about each other) can serve as a conceptual framework for exploring grassroots processes. 

Community psychology provides some useful explanatory constructs to link community perceptions and emotions related to place and environmental threat to behaviors in response to those emotions and perceived threats [50]. These place-based, but ultimately social perceptions (such as sense of community and collective efficacy) and behaviors (such as citizen participation and neighboring) serve meaning-making functions and consolidation processes in a community and constitute “social capital” at the individual psycho-behavioral level [51].

Another important topic to develop further is the link between local and supralocal levels of activism (question three above). The rallying cry of environmentalism—“think globally, act locally”—remains just a rhetorical slogan unless there are both theory and actionable plans connecting grassroots actions to national and global concerns and movements. However, this link still remains relatively neglected in social movements research [41], where the focus is mostly on organizational links between different scales of activism. 

## 5. Characteristics of the Local and the Environment

When we study local environmental activism, we should take into account important aspects of the local, the environment, and their interaction that shape activism [52]. In this section we will review and build upon studies that have treated these two aspects; we will then incorporate their characteristics in the theorizing on our three review questions.

When discussing local activism, we refer to a local, geographically-bound community as the agent. The exact scale of the local can vary, for example by the extent of an environmental use, problem or policy-making level (e.g., [53,54]). The concept of place is very useful here. According to Agnew [55], a space becomes a place when it has a specific location, a locale—the shape of place defined material boundaries and everyday activities, and a sense of place—the attachments of people to the place. The local community therefore is the group that shares lived experience in a locale, thus defining it as a common place.

Spatial proximity facilitates the creation of strong ties, trust, and a sense of community [56,57]; it structures social relations and institutions [55]. People living in a common locale build social networks and community relationships around family, neighborhood, school, work, religion [13]. The implications of local community for activism are manifold. First, communal lived experiences involve practices and cognitions of the good life [29]; this opens up opportunities for prefigurative [58] and NSM-type politics where a community’s activism is focused not on policy change but on protecting their way of life in opposition to an external power [22,59]. In contrast, social movements are often directed at a particular issue and policy. Second, and again different from mainstream contentious politics, local community activism has a strong interpersonal and affective basis rather than, or in addition to, instrumentally rational strategizing and political calculations. Therefore, understanding local activism requires application of community and social psychology frameworks, with concepts like sense of community and neighboring in a central role [51]. Third, as mentioned above, the life of a local community presents a totality of diverse experiences and multiple issues. One issue, the environment for example, cannot easily be abstracted into a separate cause in the way national movements focus on specific issues and policies. For this reason the emergence of an environmental goal in a community vis-a-vis other community issues (especially economic ones, but also others) is an important question to study.

The environment provides the natural boundaries and material exostructure for community relationships. When we study environmental activism, we mean the natural environment: material ambience (space/capacity and ability to pass through), sensual ambience (scenery, sound, smell, temperature), sustenance ambience (air, water, land, food), and living ambience (animals, plants). “The environmental experience” [13] is one important part of common lived experiences; the non-(human)-made materiality of a place mediates social interaction, mobility and daily routines [60]. But what is the meaning of nature in local environmental activism? First, nature cannot be reproduced by humans and changes in it are often irreversible [11]. Fragility and uniqueness are commonly ascribed as characteristics of the natural environment [61]. Then, nature is also a complex system, and even more complex is its relationship with human systems. As Johnson and Frickel [40] observe, “environmental problems are understood in terms of highly complex ecological and social systems interaction across multiple temporal and spatial scales” [40] (p. 320). Finally, in contentious politics terms, nature is an affected party: claims made by opponents have effects on nature and effects on nature mediate the effects on opponents’ interests. This position creates a qualitative difference in the way problems, causes, interests, and solutions are framed and understood. The fragility and uniqueness of the affected party sometimes creates a sense of local ownership, a moral obligation to protect, to act on behalf of voiceless nature [35,62]. The complexity of nature as affected party begets ambiguity with regard to the causes and effects of disruptions in nature. This ambiguity is a major hurdle for local environmental activism [41] and another potential area of contribution for environmental psychology and other cognitive sciences.

### 5.1. Place as the Interaction of Local and Environment

The local and the environment as explored so far are quite like the social (community) and material (nature) dimensions of a place. From Agnew’s definition of “place” is evident that it is both a material and a socially constructed reality. Places are “sites where people live, work and move, and where they form attachments, practice their relations with each other, and relate to the rest of the world” [60] (p. 161). The use of place as a concept will help explore local environmental activism from a place attachment perspective [63]. Two important interactions of nature and local community are local *vs*. expert knowledge and the disruption of the quotidian.

Nature as an issue for local activism presents ambiguities with regard to causes, processes, and effects of environmental disruption. This ambiguity is often deepened by the fact that environmental disruption is produced by the use of complex technologies, resulting in varying levels of threat complexity [40]. Ambiguity in itself might be advantageous to the opposition of development if considered under the principle of precaution which puts the burden of proof of innocuousness on developers [26]. While this principle has rightfully been touted as a major achievement of the EJM, the pro-business climate of the recent decades has all but nullified its application. Consequently, experts are summoned by proponents of development to testify about the innocuousness of technological intervention, or more likely, about the dubiousness of claims about harm. Local grassroots activists are in a difficult situation where their claims are pitted against “scientific” knowledge and notions of certainty [29,41]. Interpretation of technology and its fit with the local environment becomes an important task of local activism [62]. The community’s advantage comes from local knowledge. Local knowledge is based on everyday life and social interaction in a locale [26,48]. This first-hand knowledge gives authority and legitimacy to the locals to contest pro-development expertise [34] and can be seen as complementary to scientific data and thus adding clout to local environmental and public health efforts [64]. This is very different from the approach of national environmental organizations that base their claims on scientific and universal knowledge in order to influence policy-making, and often shun local protective efforts in order to protect their own credibility [29].

When local knowledge is confronted with threat ambiguity, grassroots activism can be boosted by a mechanism called disruption of the quotidian [65]. This is the disruption of everyday practices, and the expectations people have for their perpetual reproduction. A disruption punctures people’s routines and the way they take for granted their environment. The result can be grievances that beget a movement mobilization. This social movement concept fits particularly well to local environmental activism because three of the four types of events that Snow and his colleagues identify as breakdowns of the quotidian are community disasters, actual or threatened intrusion into culturally defined zones of privacy and control, and changes in taken-for-granted subsistence routines [65]. These events are very likely to have either their source or a clear expression in the natural environment. Snow *et al* [65] think that in the case of disruptions of everyday life activist framing of the issue is easier because it is “experientially commensurate”. In contrast to activism around social issues where the identification of an issue as a problem is an early major challenge for activists, local environmental activism can look to nature as the wall where the message is written. The experience of an environmental problem is a crucial element of local knowledge as opposed to abstract expert claims. However, there are limitations to this knowledge: while environmental disruption might be often palpable to dwellers, in many cases, like slowly-building air or water pollution, threat is not apparent [40].

To summarize, the ambiguity of threat can be conceived as having three aspects: (1) complexity—how difficult it is to describe the links between technological intervention and local effects; (2) actuality—whether the intervention is already done or proposed/potential; and (3) naturality—whether the intervention has a material expression in the natural environment. The combination of these aspects shape and limit the reactive environmental discourses in a community. If complexity is low and the threat is potential, discourses might be centered on development dilemmas, compensation and local control. If the threat is actual but complex, local *vs*. expert knowledge becomes a focus of contention. Actual and clear threat might result in justice, health and compensation topics. And in the particular case of potential and complex threat that has a natural expression, a community might resort to preservationist, “out-of-place” arguments and identity-based opposition. It is quite possible that environmental discourses are taken up by community members to protect economic interests because the naturality of the intervention provides opportunities to react in environmentally-protective ways without necessarily caring about nature [34].

### 5.2. Place-Based Power Inequalities

Local environmental activists might frame injustice in terms of the power inequalities of class, race, gender, or modernity. These dimensions obviously produce unjust outcomes not only for the environment where marginalized communities live. The importance of locality and place though is also in that they produce their own, place-based inequalities. The latter add an additional layer to local environmental activism that cannot be directly subsumed under the rights expansion path of social movements. We will touch here on remoteness, sparseness, mobility, and rurality as aspects of spatial vulnerability.

Remoteness and sparseness are sources of power inequality due to the “scalar spatiality of power and authority” [60] (p. 159), the hierarchical nestedness of state power. Thus decisions for development are made on different scales from the national through the regional to the local. Remoteness means a community is far from a decision-making institution’s physical base; therefore, presenting concerns or staging disruptive protest action is difficult. Additionally, remoteness also makes harder appeals to potentially sympathetic audiences. Finally, often communities that are remote from urban centers are also underserved, which makes them vulnerable when double-edged outside opportunities for development appear [34]. Sparseness, which is slightly different from remoteness because it entails living away from other remote communities as well, is a source of power weakness because of the difficulty of mobilizing the support or cooperation of similar communities, a common strategy of local environmental activism [13]. To close this point, it is worth noting that trends of power and resource decentralization discussed above, coupled with advances in communication technology, might partly alleviate place-based power inequality from these two sources.

Mobility is another place-related source of power. It is the ability to move a value in and out of the particular place. Capital and labor are two often studied values pertaining to power in place [66]. Usually, capital is movable, and labor is place-bound, which creates vulnerability and dilemmas where people choose between development and environment [19]. If capital is place-bound, the community has a higher degree of control over it, and in the case of a development that disrupts the environment, local people can better assert their interests. If the natural environment of a place represents a unique or rare unmovable resource, capital again must negotiate the terms of development with local communities and their notions of good life. However, mobility as a function of material and cultural resources also creates power inequalities within the network of an environmental movement [57] as more mobile activists becomes bridging figures and leaders. Mobility is obviously related to remoteness, when the movable value is citizens’ voice or physical presence (like in disruptive action). Mobility as an asset or use of a place is opposite to remoteness—if a place is important for its flow capacity, its community has more power stemming from the ability to disrupt this flow.

Finally, rurality can be a source of power inequality as a facet of the modernist axis discussed above. One of the elements of modernization is urbanization, supplanting the rural way of life with the better or more efficient urban order. The rural is socially constructed in opposition to the urban (and vice versa), where government actions are those of interfering urban elites [22]. The position of the rural as the periphery of the urban is culturally inferior and thus unequal in power. Variations of culturally determined power inferiority are places or whole regions that are for any reason deemed stigmatized, less deserving or backward. 

The import of placed-based power inequality becomes clear when we consider the formation of oppositional identity in response to disturbance of place. Class or race identity indeed has mobilizing force because communities frame environmental injustice in the terms of discrimination and rights. Class and race also provide opportunities for solidarity and expansion of support, while paradoxically having potential for within-community divisions based on these identities. In contrast, placed-based power inequality can produce place-based oppositional identity bridging other (divisive) identities within a community, via an appeal to “the shared predicament of living in…remote, underserved, and marginalized locales” [34] (p. 174). In other words, a realization of a common fate of being discriminated because of the place you live in is one significant impetus for the emergence of a place identity that in turn can consolidate a diverse community around an environmental protection action (cf. the second review question) [67].

### 5.3. Powerlessness and Vulnerability

Since the start of EJM the social production of unequal environmental outcomes has come to the center of environmental thinking. To reiterate, power inequalities across communities due to class, race, cultural or other differences produce differential environmental gains and losses; communities targeted for environmental risks or harms are chosen due to their powerlessness [15]. The central notion of power should be examined more critically to take into account how it performs with regard to the environment and the local community. In this section we will differentiate between a legalistic notion of rights, an informal notion of power, and the notion of vulnerability as capturing in different ways the ability of a community to resist environmental disruption.

Reform environmentalism was about new rights embodied in new laws—rights to clean air, clean water, a healthy environment. Laws empower communities by defining what they have the right to claim from governments and corporations. However, Schlosberg [11] critiques environmental justice theorists’ legalistic notion of recognition of communities as parties to environmental politics. In their perspective, recognition is a precondition to justice; we talk about rights and justice when we have citizenship (recognition). Schlosberg sees recognition not as formal and given, but as a contested element of environmental justice. A “legal right to act” is not the “power to act” [68] (cit. in [69]). The Habermasian notion of formal prerequisites of equality among deliberating opponents leading to reasoned decisions may conceal realities of unequal capital. While a legalistic problem persists, where certain communities are still not recognized as full citizens due to power inequalities when environmental laws are enforced, as our earlier discussion showed, there are other reasons that power will remain important above and beyond rights in a formal sense. First, the state consistently privileges growth and capital accumulation [2,14], so the regulation of an industry is skewed in its favor, often referred to as “regulatory capture” (for example, corporations can deliberately take risks, pollute and pay fines without being criminally prosecuted) and sometimes even rolled back to stimulate development (as in the exemption of hydraulic fracturing from the Clean Water Act). In this hypothesis, corporations are back in the situation described by Gibbs [15] where they pick communities with less power to resist. Second, even in a world of perfect formal power equality across communities, the ambiguity of the link technology-nature-human health renders rights all but irrelevant. In the case of toxic waste the harm, the cause, and the rights of the affected were unambiguous; later issues such as incinerators [70] or more currently, hydraulic fracturing, carbon capture and storage, or underground coal gasification, do not have immediate and clear effects on nature and human health. Rights cannot be invoked to stop such projects because the effects on nature and communities are place-specific and contested between experts, corporations, bureaucrats, and citizens. Consequently, a community’s informal power becomes crucial in the decision-making process.

Even this informal notion of power does not capture all the subtlety of power inequality. We introduce the notion of vulnerability and compare it to powerlessness. Consider as an example the difference between a community sitting on a toxic superfund site and a community that is approached for a fracking operation. The former situation is unambiguously coercive and unfair: the risks are clear, the harms are visceral; for the community the site is a harm and only powerlessness can prevent action against the environmental disruption. In the latter situation, the damage is unclear and conditional on technology and its reliability. There are also gains in the form of royalty payments to local land owners. In this case, whether the community will accept environmental threat or fight it depends, among other factors, on its affluence, self-sufficiency, or better alternatives for development. The absence of such local and locally-controlled assets we call vulnerability. Vulnerability means that a community, in a situation of a trade-off between absence of environmental risks and opportunities for economic development, may choose one “good” at the expense of another (employment *vs*. health). As Saitta succinctly put it, “wages were more important than health” [19] (p. 1302). Poverty, as a measure of the lack of locally-controlled assets, has been related to environmental destruction [17,19]. Not surprisingly, as we noted above, ingenious environmental rights are most often a claim for a locally-controlled modernization [21] as opposed to “industrialization without development” [19].

An analysis of different forms of power can show the distinction between powerlessness and vulnerability: three basic forms are “power from”, “power to” and “power over” [71]. Communities opposing intrusive development attempt to assert their *power from* imposed decisions (corporations or states exercising “*power over*”). They do so because they have the *power to* pursue their own vision of a good community, with the resources needed to accomplish it. Poor or marginalized communities that do not have this *power to* may prefer to accept externally-driven development, and not seek *power from* the force that promotes it.

In addition, issues of powerlessness and vulnerability pertain not only to the relationship between community activists and forces that threaten the environment. Power inequalities exist within environmental movements, across communities and activists. A local grassroots group often finds itself in a marginalized position within a network of activist communities, and the main determinant for this is mobility, in turn determined by material and cultural resources [57], or *freedom to*. Such marginalized position means not having voice in the strategizing, steering, and resource spending of a networked movement, which might mean irrelevant or adverse movement goals and outcomes.

## 6. Environmental Discourse Emergence

### 6.1. Approaches to Analyzing Environmental Discourse Emergence

Our first review question pertains to the onset of local environmental activism: How does the environment emerge as a topic for community activism and how a particular environmental discourse becomes dominant? The environmental literature tends to give more general, descriptive answers. For example, in the global North the environment is seen as a prominent social fault line where popular struggles coalesce, while in the global South it competes with human rights and democracy [29]. In another perspective, in industrialized countries where industrial development is internally-directed and long established (“primary”) the environmental discourse is focused on health and trust in technology and the experts; in developing countries where development is externally-driven and novel (“secondary”), the discourse is focused on poverty and vulnerability, rights, local control and participation [19]. We accept these observations as valid and probably applicable to the study of local activism, but we attempt to address the question at the community level, following Bonaiuto *et al*’s recommendation that “pro-environmental attitudes (…) should be conceived as place-situated phenomena” [53] (p. 634). Our goal is to show how environmental psychology concepts and research can enrich social movement theory and social representations theory as approaches to environmental discourses, not in mechanistically predicting a particular discourse in a particular place, but in pointing to links worth exploring.

**Figure 1:**
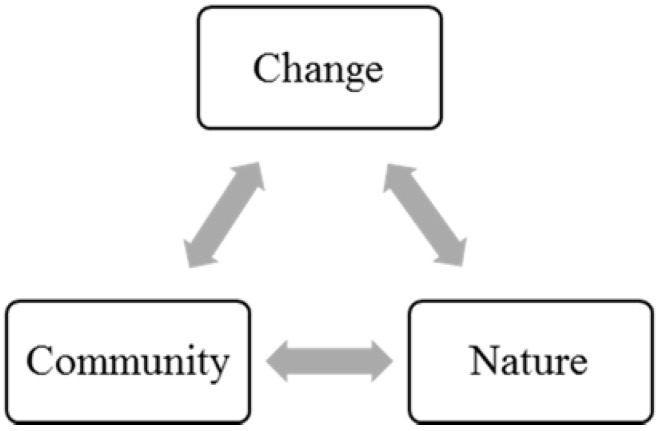
Community interpretation of relationships of change to nature within a place.

When we discuss the emergence of an environmental discourse, we refer to the community processes of interpretation and communication of an actual or imminent disruption to a place and its natural materiality [48,72]. To build on our analysis so far, we start with a general model with three elements in a place, whose relationships are objects of interpretation by local dwellers and activists as they make sense of a place change (Figure 1). 

The change in place is a potential or actual event/process that reconfigures the material and social aspects of a place. At this point we do not evaluate it as a disruption, threat or development—evaluations are an outcome of interpretive community processes [48]. Members of the community make sense of the relationship of the change and its agents to the local community via social interaction, communication and relating to the community’s history and self-image. These interpretive processes can facilitate the emergence of an *environmental justice and rights* discourse or *local control* discourse if the community relates this relationship to history of discrimination [4], a consciousness of power inequality [15,34] or an unfulfilled expectation of a just—that is, participatory-democratic—decision-making about the introduction of change [11,48].

To apply an environmental psychology lens, we dwell mostly on the other two interpreted relationships and how they shape community discourse and reaction to change. The links between the community and its natural environment can be conceptualized as community place attachment: the cognitive (identity), affective (bonding) and behavioral (dependence) attachment of individuals and communities to a place [47,73] in this analysis, to the natural elements in a place. From a social representations (SR) perspective, we can talk about the community representations of nature, comprising identity, cognitions and practices of human-nature relationship [74].

The link between the change and nature within a place is interpreted along or compounded with the interpretation of change to the community-nature *relationship*. Major topics of interpretation here are technology, risks and (local) knowledge. Recent SR approaches in environmental psychology [48,50] examine disruption with a focus on the perceived and symbolic dimensions of place, and the “fit” between the new development and existing place attachments and place meanings.

Before elaborating more on the two relationships we will, for purposes of comprehensiveness, point briefly to the context of the tripartite model as a source of discursive material. A community’s interpretation and communication of change happens within a larger discursive context of the environmental sector and society as a whole. The dominating discourse of the time, be it health [10], rights and justice [4,12] or global climate threat [41] is likely to shape local discourse if the particular community activists want to bridge their struggles above the local level.

### 6.2. Interpreting Nature and Change, and the Emergence of Environmental Discourses

We will examine place-framing, social representations, and place attachment theory for their current and potential contribution to the explanation of interpretive community processes.

Place-framing analysis is an application of framing theory from social movements [46], focused on “the material and symbolic dimensions of place as a basis for collective, identity-based activism” [34] (p. 174). Martin [35] used directly the three functions of frames [46]: motivational (characterizing the community as activist), diagnostic (defining problems and assigning blame), and prognostic (defining solutions and actions to achieve them). The latter two functions are most closely related to the interpretation of environmental change. However, how place features in these frames is not fully elaborated yet in the place-framing model. A proposed or actual change in the palpable natural environment of a place can *objectify* change—a SR cognitive process of making the unknown known by “transforming it into something concrete we may perceive and experience with our senses” [75]. Just as important, the boundaries of a locale [55] provide a material criterion for defining in *vs*. out and thus may facilitate the crucial realization of individual *vs*. structural causes of the issue. It is worth exploring these cognitive uses of place and nature in diagnostic framing. In terms of prognostic framing, Martin [35] studied changes to the physical place as proposed solutions to a local issue. We suggest advancing this idea by exploring a community’s representation of ideal community and how it relates to actions and goals. An ideal community imagined in the past can give rise to a reparative or preservationist discourse; an ideal community imagined in the future can facilitate a creative, developmental discourse.

What these processes of framing are meant to do is essentially explanation, or making something unknown known by connecting it to existing knowledge [76]. This connection work is known as frame alignment [77] in social movement theory, but its cognitive and communicative mechanisms are not as well elaborated as in SR theory [75]. This is why we believe a SR approach is very useful (but still underdeveloped)—it can study the relating of existing collective representations of nature and community-nature relationships to the new and unfamiliar reality of environmental change [74]. Furthermore, nature should be studied as an opportune subject in SR theory because scholars in adjacent fields like cognitive linguistics [78] claim that metaphorical thinking (the basic way of knowing and learning) uses spatial and natural anthropocentric relationships to make the world comprehensible [79].

While we claim that the SR developments in environmental psychology have advantages over the framing approach, we need to elaborate them more to be able to explain local activism. We unpack place attachment and the symbolic meanings of place [48] and relate them to the emergence of the environment as an issue and a particular environmental discourse in a community.

#### 6.2.1. Place Definition 

Place attachment will be used in this paper as a multidimensional concept comprising behavioral, affective and cognitive elements [73]. Place definition is “the socially constructed and negotiated boundaries of the place, and the features and attributes of the place that give it a distinctive identity in the minds of dwellers” [50] (p. 65). It is not part of place attachment per se, but is a precondition for attaching oneself to a particular place and is relevant to the emergence of environmental discourses in two ways. First, the prominence of nature in dwellers’ definition of place predicts the emergence of environmental concern. Some studies examine natural as opposed to social representations of place [80,81]. Strong connectedness to a place’s nature predicts proenvironmental behaviors and attitudes [82] while attachment to the social aspects of a place correlate with pro-development attitudes [81].

Second, and even more important, is the cognitive representation of nature in the minds of local community members. For example, nature defined as fragile and unique was associated with preservation and restoration discourses among the dwellers of Louisiana coastal communities [61]. Opposition to airports expansion in the UK was framed in environmental justice terms except in a community with unique nature where preservation was the dominant discourse [41]. We want to generalize these observations by proposing to think about a place-based ontology of human-nature relationship [74]. Taking from the history of environmentalism and environmental discourses, we can posit that there are two important dimensions of this ontology: whether community members conceive nature as part of, or as apart from community [29], and whether they conceive it as equal or subordinate to the community. Instead of transposing the already discussed discourses to the community level, we continue with place attachment concepts in order to show how these cognitions are shaped by behaviors, emotions and identity in place. 

#### 6.2.2. Place Interdependence

Place dependence is established as the behavioral dimension, or element, of place attachment. It is defined as a functional connection between an individual and their physical setting [83], indicating how well the setting serves an intended use [84], *i.e.*, users depend on it for certain needs [85]. This understanding of the concept is very similar to the idea of the sustenance quotidian and its possible disruption (see above). Elsewhere [50] we proposed the concept of place interdependence to better capture the entirety of human behavior in a setting: the dependence relation between the individual and the place is not one-directional; people create the place, including its natural configuration [74], they exploit and/or take care of a place. The influence of an individual over a place is related to cognitions of the place as an extension of the self (a dimension of place identity for Droseltis and Vignoles [86]), a sense ownership of the place (identity-related symbolic meaning), or investments in place (functional meaning).

Studies of interdependence of locals and nature suggest that environmentally-related attitudes and actions emerge when the current human-nature practices, nature use and nature enhancement are disrupted by change. In a study of attitudes toward industrial development of renewable energy Devine-Wright and Howes [87] found that resistance with a preservation discourse arose from the contradiction of environment providing restorative benefits and a new industrial use that “fences the bay”. Another preservation discourse example comes from Woods’s study of rural activism [22] where residents who had commodified local nature for tourism and lifestyle purposes opposed resource-extraction projects. In contrast, Appalachian Trail hikers perceived favorably development on the Trail as it enhanced their use of nature [88]. A very interesting example is found in Bonaiuto and colleagues’ study [53] where some local residents (labeled ‘economists’) opposed environmental protection of a natural area because of their commercial, extractive practices in the place. The logic of these cases relates to Dunlap’s conceptualization of environmental problems as results of conflicting functions (uses) of nature in one area: supply of resources, sink for waste products and habitat for living [89].

We propose a more general way to think about place (and its nature) interdependence and its relationship to environmental discourses. Applying again ideas from the history of environmentalism, we suggest a two-dimensional model of nature practices/nature interdependence (Table 1). The first dimension is the familiar dichotomy of nature as part of community’s practices and as something apart from them. The other dimension is active *vs*. passive behaviors toward nature—the former meaning transformative, shaping the natural environment.

**Table 1:** Nature interdependence and environmental discourses.

	Nature Apart	Nature as Part
Passive behaviors	Commodifying	Ecological
Active behaviors	Extractive	Organic

We label practices “ecological” when nature is part of a community’s life, but only as environment sustaining human life, taken for granted. Usually this situation is only questioned in the case of health-threatening pollution. Communities with such nature interdependence are likely to engage in health and risks discourses. “Commodifying” practices treat nature as a backdrop for a desired lifestyle. Its main use is as a commodity, related to property values, “a rural idyll” [22] lifestyle, or tourism. It is the perceived untouchedness of nature that is valued (hence a passive behavior). Communities (or members) with such interdependence are likely to engage in preservationist and local control discourses. Practices that are both changing and treating nature as apart from humans are “extractive”—using nature as a source of raw materials is an example. If this is a dominant practice in a community, we are likely to expect local control (if a competing outside project is proposed) or conservationist discourses. Finally, a behavior that is both active and one with nature is “organic”—these are often traditional lifestyles where communities’ sustenance practices are rooted in nature (e.g., fishing, farming) and they see their life as a harmonious part of a natural balance [74]. When this particular human-nature relationship is disrupted, discourses of preservation, indigenous rights and knowledge, or deep ecology are likely to emerge.

#### 6.2.3. Place (Nature) Bonding 

The affective component of place attachment is place bonding—individual or community positive emotional ties to a place. In PA theory a further distinction is made between nature bonding and social bonding—affective-emotional bonds to the natural environment and to friends, family, community respectively [50,73]. Place bonding has been associated with a negative attitude to place disruption [87]. It is not difficult to predict that strong nature bonding would predict the emergence of the environment as an issue. We want to make two conceptual steps further: first, differentiate between positive place affects instead of treating place bonding as continuous from positive to negative; and second, examine links between these categories of affects and corresponding cognitions and behaviors. Place attachment is not just a mechanistic sum of its elements; can we differentiate between positive affects related to nature and how they relate to specific human-nature cognitions and behaviors in place? An affirmative answer is also supported by SR theory, where SRs have value, emotional, cognitive, and behavioral elements [75]. For example, positive emotional ties with nature might involve pride, appreciation, care, satisfaction, or dependence. These emotions might be related to particular nature cognitions, behaviors, and identity. Appreciation or care might be in a cluster with organic interdependence and a cognition of nature as one with humans; pride might be coupled with restorative and preservation discourses; satisfaction might relate to commodifying or extracting practices; and taken-for-granted dependence could go with ecological cognitions and behaviors (refer to Table 1). Even more interestingly, we can borrow more from SR theory and examine emotional dichotomies [75] as anchoring mechanisms for making sense of imminent natural disruption. If positive and negative affects are dichotomist, we can think about how nature bonding transforms into natural change *aversion*, and then test for the correlation of particular negative affects to particular environmental discourses. For example, social psychology research suggests that sense of injustice (and hence justice and rights discourses) is associated by emotions of outrage rather than pity [90]. Can we draw connections like pride (positive)—shame (negative)—local control (discourse); appreciation—outrage—justice; dependence—fear—health/risks; and satisfaction—frustration—restoration, reparation and local control? These links, of course, are not yet backed by enough PA research but we think they are worth exploring as they provide a more detailed treatment of the place attachment concept and a more organic examination of change than the “fit” approach.

## 7. Community Consolidation around Environmental Discourse

### 7.1. Place Identity

We began with a critique of some scholars of local environmentalism for reifying a unitary community as an agent: a community that understands environmental issues as one, articulates interests as one, and ultimately acts as one. Instead of propagating such assumptions, we state as a second central question, how does a community become consolidated around an environmental issue? If a plurality of environmental discourses has emerged from the relationships between community, nature, and change, how a discourse becomes consolidating?

Within-community difference might be based on the same social diversity that creates between-community discrimination: class, race, gender. Differences can also be more place-related, growing from different place cognitions, place interdependence (including economic interests in nature) and place bonding. A crucial discursive and interpretive process in local environmental mobilization is the creation of a common identity that enables a community “to bridge dissimilar environmental values and practices” [34] (p. 172). And the main pathway to this common identity in the framing literature is to “prioritize place over other social identities” [35] (p. 733). Identity framing is part of the third (and last) framing process, motivational framing, in social movement theory [46]. Place identity is also the last, cognitive, element of place attachment [73] and we will discuss how these analogous concepts from different disciplines can communicate to answer our question.

Rural studies applying framing theory to place identity start with an overall observation that place identity has largely supplanted occupational identity as a consequence of neoliberal decentralization, devolution of government, and unraveling of corporate group representation [34]. Groups with diverse social and economic statuses living in the same place are more alike than groups of the same status residing in different places. This observation is consistent with the first source of common place identity that place framing scholars advance: common identity is based on common daily-life experiences, sights and conditions that foster “location-based commonalities” [35]. This idea in itself does not give us suggestions about how a community becomes consolidated because commonalities of place are definitional. Another, more elaborated source of common identity in place framing studies is power conflict and what we articulated previously as place-based power inequalities. Within this view, a community is consolidated in resistance to an outside force. Locals realize they share a position of power weakness; this power inequality may be place-based, as in the case of rural communities. Stronger outside pressure results in stronger common identity [34]. This consolidating mechanism is consistent with Gamson’s [91] perspective on framing, which has injustice, agency and identity elements. For Gamson, activist identity is defined in opposition to an adversary (oppositional identity was adopted also by NSM theory). The in-out dichotomy inherent to place seems to facilitate adversarial framing. Rural is contrasted to urban in place-framing studies [22]. The awareness and articulation of a place-based power inequality also contribute to injustice framing, which is based on moral indignation [91]: rural communities only want to live the way they like, but are encroached and threatened by globalization, state bureaucracies and corporate greed [22]. The result is a consolidating frame with libertarian, NSM and “rights” elements.

The place-framing perspective is thus focused more on the identity consolidation process than on how exactly the materiality of place features in the resulting common identity. The adversarial framing, albeit facilitated by the physical boundaries of a place, is a process germane to any, not just local, activism (oppositional identity was adopted broadly by NSM theory). Furthermore, this scholarship addresses less emphatically some questions it initially posed itself: how are reasons and scope of activism articulated within identity framing [34,35]. In other words, why are we the ones to act and how is this related to place (nature)? In Martin’s study, these questions are answered in place-unrelated terms: we have a responsibility to act, we are a family-oriented community, we are workers [35]. Identity in the place-framing studies seems to be place-based, but not so clearly or explicitly place-related.

Environmental psychology, with PA and SR theory, can complement place-framing by its focus on the contents of place identity. In PA studies place identity is examined on an individual level as a predictor of reactions to place disruption, and not as a collective outcome (or a mediator) of local activism. Place identity is defined as “a cognitive mechanism, a component of self-concept and/or of personal identity in relation to the place one belongs to” [92] (p. 281). “The interpretation of the self would use environmental meanings to symbolize or situate individual identity. Thus one’s identity can be partly formed, maintained and transformed in relation to features and uses of everyday environment(s)” [93] (p. 160). Place identity is related to negative attitudes to environmental change provided that change is interpreted as antithetical to a place (e.g., [87,88]). Place identity also seems to be associated with pro-environmental attitudes [92]. More certainty about this relationship is hindered by inconsistencies in place identity and place attachment conceptualization [92]. We can hypothesize that community consolidation around an environmental cause will be more likely if nature features prominently in a community’s place identity. From a social representations perspective we attempt to look into the contents of place identity (as with place interdependence and place bonding): what is the interpretation of self in relation to the natural surroundings. Conceptual inconsistencies actually hint to possible modalities of place identity: while some authors study place identity as a general concept, others do in its relation to natural surroundings, natural resources or as landscape attachment [92]. Consistent with our discussion of place definition, place interdependence and place bonding, we posit that different place identities will be rooted in different community-nature representations, different self-articulated community roles in relationship to nature. Examples of identities that can be gleaned from studies in this review are nature beings, nature owners, nature users, nature knowers, global nature protectors, guests, witnesses (e.g., [34,61,74]). Threats to nature become threats to how one defines themselves [94]; for example, the incremental loss of wetlands (and thus markers for navigation) in Louisiana was experienced painfully by local navigators as destruction of personal identity and life purpose: of local knowledge, authority and agency [61].

### 7.2. Place Attachment as a Comprehensive Concept

The discussion of place identity demonstrates again the relatedness of the components of place attachment and the usefulness as a comprehensive concept. Place identity in relation to nature is often framed in terms of nature-related behaviors and practices, as a performative identity [95]. Similar is the logic of social representations of nature where labor has a primary role [74]. Taking nature as a more specific element of a place allows for a better observation of the human-place relationships in their behavior, cognitive, and affective aspects. Moving forward from the study of environmental change as an analysis of symbolic fit between change and meanings of place [48], we can examine change as it relates to place interdependence, an emotional bond, and a nature-related identity. A step further is to ask: can we conceptualize a dweller *vs*. an activist place attachment, or are these two forms of a same modality of human-nature relationship? When we talk about place interdependence, we acknowledge the possibility of pre-threat investments in nature; nature defense and nature maintenance behaviors might be interflowing; activism is not just a sudden awakening after a life oblivious of nature in a place. When we consider nature bonding, we can connect dweller and activist bonding via the emotional dichotomies discussed in the previous section. And can we discern a link between certain activist identities and nature-related place identities? A dweller-activist place attachment is probably more easily conceived via the use of narrative forms, an alternative framing model by George Lakoff [96]. Some basic narrative forms describing activism are “self-defense (villain hurts hero-victim), rescue (hero, with helpers, fights and wins over villain), overcoming obstacles (hero as victim of circumstance who surmounts difficulties), and achieving potential (hero has special potential and, through discipline and fortitude, achieves it)” [96] (p. 129). Such narrative forms are more holistic in that they contain representations of sets of causes, agency, roles, and goals. The framework of the hero’s roles can be easily translated into how a community interprets itself in relation to an environmental disruption.

Two important specifications are necessary in order to avoid overstatement of continuity in place attachment. First, place attachment pre- and post-threat can indeed change in significant ways, because social movement research has shown that place “acquires meanings through campaigns, and communities forge identity even as they mobilize against threats to their survival” [29] (p. 722). Indeed, place attachment can be sensitized by threat [48,97] and identity is both a process and a product of activism [98]. Second, PA components might be affected in different and contradictory ways by change, for example by enhancing place interdependence while impairing place identity [88]. Such contradictions are well elaborated in SR theory, within the concept of cognitive polyphasia. Further research is necessary to examine continuity and disruption in place attachment and identity and the consistency of place attachment components in relation to environmental change at both the individual and community levels [99].

## 8. Local and Supralocal Environmental Activism

### 8.1. Local Activism and NIMBY

The third question we posed was: What is the relationship between local and supralocal activism (regional, national, global)? The issue of detached and protective *vs*. expansive and transformative local environmental activism is an old one in planning, environmental, political and psychological literature [100]. We will begin by describing shortly the NIMBY concept as an analytical tool to discuss local-supralocal relations and then present some possibilities for expansion of local activism.

“Not-In-My-Backyard” is a genre of activism that, despite its personal perspective of notation, has mostly been used by outside critics. It refers to “protectionist attitudes of and oppositional tactics adopted by community groups facing an unwelcome development in their neighborhood” [48] (p. 430). This activism is criticized by politicians or planners [101] for several reasons. First, communities are accused of selfishness, because they oppose a necessary development for the common good (e.g., a landfill), a locally unwanted land-use, LULU [69] that is however publicly desired. Second, NIMBY groups are depicted as ill-informed, ignorant, irrational and alarmist [48,69,102]. We can see that these two characterizations depict NIMBY activism as anti-modernist [103] from the modernist perspective of rational, science-driven, national-scope institutions. Other criticisms claim that NIMBY activism is driven by vocal local minorities [69], which speaks to our second review question. “NIMBY” can even be simply equated to “local”, when a community remains in its actions within its territorial and issue boundaries [104].

The NIMBY concept has been criticized profusely by scholars of local activism. It has been evaluated as being a political label instead of an analytical characterization, stuck by politicians and developers whose plans are obstructed by local resistance [101]. Researchers have also demonstrated that local activists are highly informed about risks and technology and that self-interest explanations glorify rational choice and ignore the importance of issues like justice, power, and trust [48]. 

The NIMBY accusation has also become very ambiguous with the decline of modernist pathos. With decentralization and devolution of power under the optimistic label of governing through communities, NIMBY activism may be praised as grassroots democracy and empowerment [69]. Pro-development agents have faced the difficult task of differentiating “good” and “bad” participation [102]. What was seen in NIMBY as anti-modern parochialism is now framed as tensions of postmodernity: a crisis in trust to experts and science, a disillusionment with democratic deficits and accountability [105].

While the NIMBY label has been criticized for the political tool it sometimes is, and the phenomenon itself has acquired new nuances in post-modern societies, it is a matter of discourse and contest around environmental change. When we discuss expansion of local activism, we should bear in mind that a community might focus on symbolic work of expanding their cause not for reasons directly related to the change, but to avoid the NIMBY accusation. The NIMBY label can be almost as undesirable as the local disruption.

So far in this section we have demonstrated that a local environmental protest does not have to become supralocal to satisfy certain expectations. Indeed, often the institutional framework of an issue leaves power entirely at the local level and scaling up is irrelevant. What we are interested in though is expansion of local activism when change at the supralocal level has a liberatory potential. This is the case when a policy change empowers or secures other communities as well (“Not In Anyone’s Back Yard;” e.g., environmental rights laws) or because it addresses the source of the problem [15,69]. Examples of the latter are regulations and restrictions of technological processes that produce environmental problems. The liberatory potential of such actions is in that their goals secure communities’ *freedom from* environmental threats and unjust pressures. Expansion of community activism can also bring about *freedom to* control their environment and local development, to defend a desired way of life. Such expansion above the local level is expressed in building awareness of issue causes, solidarity and common identity with other communities, networks of organizing capacities.

### 8.2. From Local Activism to Social Movement

The social movement literature takes serious interest in how particularist action transforms into a social movement. Flacks [106] talks about how resistance movements, which can be reactive and protective, become liberation movements striving for radical change. Dalton and Kuechler [107] describe the transition as one from interests to ideology. We can see that these descriptions are based on behavioral and cognitive changes respectively. These dimensions are helpful in discussing types of local environmental activism expansion.

The first scenario we see in local environmentalism studies is *political expansion*. Transcending the local is necessary because the issue must be politicized and decided on a national policy level [41], turned from a routine planning dispute (where locals are pitted against experts and bureaucrats) into a high profile political issue [41]. Local activists attempt to frame the threat they face as pervasive and universal in order to attract public attention and put it on the political agenda [40]. Issues salient on the national political agenda can be taken up by environmental movement organizations (EMOs). Another path to putting pressure on the national policy level is through networking and coalition-building with groups with similar grievances and coordinating a national or regional challenge [29,44]. In the political expansion, framing work is very important to relate to the general public, not just to define the issue as public, but also to present the challenge as just and legitimate. Sometimes local issues must be strategically framed to align with current master frames such as justice or climate change [41] or risk being left in public oblivion [10]. The role of regional and national EMOs is also crucial, as intermediaries between local and national scales and as “experts” in policy advocacy [108]. The work of local and national organizations can be complementary within a division of labor along the policy-making process, as when local activists highlight an issue that national organization then present on a political level [29] or competitive, when national organizations pick issues or goals and carry the rewards of success [57]. The political expansion is consistent with a contentious politics perspective on social movements (e.g., [109]) where challenges are made to governments via institutional and extrainstitutional means with the goal of policy change.

A second scenario described in the environmental literature is *grassroots expansion*. This is exemplified best in the anti-toxics movement [15]. Emerging local communities that frame their issues in power inequality and environmental justice terms learn about each other and connect into a network [13,26]. As justice is the unifying frame, the community environmental problems can vary. No umbrella organizations or advocacy EMOs are necessary; in the case of the anti-toxics movement, the Citizens Clearinghouse for Hazardous Waste (CCHW) was founded by Lois Gibbs as a platform for sharing experiences and connecting local activists. A community-organizing model for expansion was established where new groups mobilize and contact CCHW for training and consultation while they maintain full local control of their strategy and actions [26]. Such a movement is a network of places [57], where local organizations are affiliates that adopt similar discourses, claims, and tactics, but do not follow a grand strategy or the orders of social movement organizations [57]. The links of solidarity and identification are strong, but the lack of resources and disparate issues and timing do not allow for the creation of strong ties and structure among organizations [57].

*Cultural expansion* we call links between a community and the outer world based on awareness of larger issues and identity. This expansion fits best with NSM perspectives on social movements, where policy impact and national organizations are irrelevant; instead, groups share oppositional identity and the desire to create alternative ways of living to bureaucratic rationality [7]. Some authors qualify local activism as non-NIMBY even only for reflexivity and understanding of power and issues beyond the local [29]. NSM local environmental activism does not have to have a common goal, target, or action. Such groups strive to be left alone, to define and construct their own identity and life, alternative and in opposition to hegemonic norms (prefigurative politics). Local activism exists in a loose cultural network, for example, rurality [22]. Communities that oppose externally-imposed development understand the larger conflict between rural and urban/post-modern and modern, and they share oppositional codes and loosely defined identity; but they do not cooperate in networks or organize for national politics because their fights are local and defining them in their own way is part of the struggle [34].

Finally, we should acknowledge a type of expansion we term *tactical*. Its purpose is to gain support and sympathy of outside publics or obviate NIMBY accusations. Because the NIMBY label has generally become so undesirable, local environmental groups can adopt larger frames such as justice or preservation in order to legitimate their opposition to a local development. The environment actually presents an opportunity to frame a concern in a less self-interested way because, as we discussed previously, nature can be an affected side apart from the claim-maker. Thus rural activists equated preservation of rural lifestyle to environmental preservation [22]. Similarly, an environmental rights framing was applied by cyclists in London who demanded bike lanes from the city [110] in order to gain public support. Certainly, it is difficult to judge whether tactical expansion is just impression work or is a beginning of a more profound understanding of the issues. However, often such causes do not multiply or expand after their success.

### 8.3. Place, Nature and Expansion of Local Activism

Within this section we reviewed mechanisms of expanding local activism to a supralocal level: universalizing the issue, picking a national institutional target, developing a broader understanding of causes, aligning with salient societal frames, developing a common identity, networking and organizing for national campaigns. We conclude by briefly discussing a more specific mechanism germane to local environmental activism: the expansion of place and place identity. As we stated earlier, the definition of a place, including its boundaries, is an outcome of interpretive and communicative processes. Environmental psychology research suggests that different definitions have differential outcomes for environmental attitudes. For example, natural protected areas in Italy were viewed negatively by local residents while positively by citizens of the wider region [53]. We posit that a way to expand a local issue is to expand symbolically the place it affects, frame it as relevant to a wider audience. This was the case with the Gnangara Groundwater System which became an important region for the citizens of Perth, Australia [54]. A unique or very important natural area can be framed as a national treasure and thus its place of interdependence or symbolic relationships can be expanded to the national territory [93]. Communities sharing similar power and environmental inequalities, and proximity can construct a regional definition of place, such as “Up North” in the case of rural communities in British Columbia [34]. Finally, certain places can be generalized (instead of expanded), as when place-based power inequalities create solidarities and awareness of a common issue (e.g., marginalized rural communities).

## 9. Conclusions

Local environmental grassroots activism is strong and ubiquitous, keeping pace with a world where the national scale of problem-solving devolves and sublimates into the local and global. Its study must take into account complex relationships between individual and community, place and region, humans and nature, experience and technology, fate and power. We reviewed three main sources of scholarship on local environmental activism, suggested areas of contact and cross-pollination, and advanced tentative frameworks and hypotheses for future research. We have emphasized the limited but growing theory and research in environmental psychology and the already extensive literatures in environmental sociology and politics. This multi-disciplinary approach is challenging, but necessitated by the inherently complex and multi-level nature of local environmental activism—comprising characteristics of the local and the environment, of individual and community psychology, of social and institutional relations. Disciplines that, although pertinent, were not included in our review, are community psychology, where studies of local environmental issues are still inchoate [111], and the study of individual-level environmental behavior (e.g., [112,113]). We decided to focus on the three chosen disciplines as they held most potential (and actual studies as well) for a multi-disciplinary approach and new directions for research. The purpose of this review is not to exhaust all sources of scholarship, but to demonstrate how environmental psychology can add to established disciplines of contentious politics. Indeed, power inequalities and their challenging are what local environmental grassroots activism and related social movements are ultimately all about. However, this text aimed to demonstrate that environmental psychology can also contribute to researchers’ and activists’ agendas resonating with a diverse constituency of interest groups and stakeholders “to construct and politicize a local sense of place as a means of rallying insiders against outside forces and pressures” [34] (p. 172). Our paper contributes to scholarship on local environmental activism in several ways. We argued for the increasing significance of the local scale by summarizing trends in three fields of social-political development. Then, we included a discussion of what local and nature means as a prerequisite to studying local activism, with the choice of nature’s fragility and uniqueness, complexity, and status as an affected third party as crucial for understanding activism. Finally, we showed how local and nature interact in a place by elaborating on ambiguity (threat complexity, naturality, actuality) and local knowledge.

Another important prerequisite for the study of local environmental activism we advanced was place-based power inequalities. We added this dimension to the well-established axes of class, race, and gender in studying inequalities. Four sources of place-based inequalities were proposed from synthesis of prior research. The framing of place-based inequalities paves the way to study more carefully common place identity as a mobilizing force for activism. We also discussed three expressions of power inequality (formal, informal, and vulnerability).

Next, we elaborated a place-based approach to the emergence of environmental discourses in a mobilizing community. Brief suggestions for a more expansive application of place-framing were made, in the direction of including more consistently place, use social representations, and go beyond frame functions from social movement theory. Then, place attachment theory was applied to activism, above and beyond well-established research on reactions to place disruption. Activism and place attachment were elaborated in a common framework. To accomplish this synthesis, the elements of place attachment were elaborated in novel ways. Place definition, which is rarely taken into account in place attachment studies, was discussed. Next, place interdependence, a concept promoted by the authors, was advanced within a two-dimensional model of nature interdependence and environmental discourses. Nature bonding (as part of place bonding) was expanded in important ways: first by differentiating between positive place affects instead of treating place bonding just as continuous from positive to negative; and second, by examining links between these categories of affects and corresponding cognitions and behaviors. We then proposed hypotheses about how these links can work, and how they can translate into reactive (and possibly activist) affects.

When we discussed the consolidation of a community for activism around an environmental discourse, we applied environmental psychology and social representations to the study of place identity as a unifying community force. This approach gave us an opportunity to glean at the content of place identity. The next advance we proposed was to conceptualize dweller and activist identity, and place attachment more generally, as connected and perhaps interflowing. This step treats place attachment as a more comprehensive and consistent phenomenon and relates confidently place attachment studies to studies of activism. A continuous place attachment model also allows for the use of narrative frames in the interpretation of nature changes.

Finally, in the discussion of the links between local and supralocal environmental activism we proposed, based on prior studies, four types of expansion scenarios. To that we added a place-based expansion, in line with the environmental psychology approach informing our paper.
